# Metabonomic analysis to identify exometabolome changes underlying antifungal and growth promotion mechanisms of endophytic Actinobacterium *Streptomyces albidoflavus* for sustainable agriculture practice

**DOI:** 10.3389/fmicb.2024.1439798

**Published:** 2024-08-30

**Authors:** Osama Abdalla Abdelshafy Mohamad, Yong-Hong Liu, Yin Huang, Nigora Kuchkarova, Lei Dong, Jian-Yu Jiao, Bao-Zhu Fang, Jin-Biao Ma, Shaimaa Hatab, Wen-Jun Li

**Affiliations:** ^1^State Key Laboratory of Desert and Oasis Ecology, Xinjiang Institute of Ecology and Geography, Chinese Academy of Sciences, Ürümqi, China; ^2^Department of Biological, Marine Sciences and Environmental Agriculture, Institute for Post Graduate Environmental Studies, Arish University, Arish, Egypt; ^3^Department of Environmental Protection, Faculty of Environmental Agricultural Sciences, Arish University, Arish, Egypt; ^4^Faculty of Organic Agriculture, Heliopolis University for Sustainable Development, Cairo, Egypt; ^5^State Key Laboratory of Biocontrol, Guangdong Provincial Key Laboratory of Plant Resources, School of Life Sciences, Sun Yat-sen University, Guangzhou, China

**Keywords:** medicinal plants, endophytes, actinobacteria, metabolomics, agriculture sustainability, environmental microbiology

## Abstract

In recent years, there has been an increasing focus on microbial ecology and its possible impact on agricultural production, owing to its eco-friendly nature and sustainable use. The current study employs metabolomics technologies and bioinformatics approaches to identify changes in the exometabolome of *Streptomyces albidoflavus* B24. This research aims to shed light on the mechanisms and metabolites responsible for the antifungal and growth promotion strategies, with potential applications in sustainable agriculture. Metabolomic analysis was conducted using Q Exactive UPLC–MS/MS. Our findings indicate that a total of 3,840 metabolites were identified, with 137 metabolites exhibiting significant differences divided into 61 up and 75 downregulated metabolites based on VIP >1, |FC| >1, and *p* < 0.01. The interaction of *S. albidoflavus* B24 monoculture with the co-culture demonstrated a stronger correlation coefficient. The Principal Component Analysis (PCA) demonstrates that PCA1 accounted for 23.36%, while PCA2 accounted for 20.28% distinction. OPLS-DA score plots indicate significant separation among different groups representing (t1) 24% as the predicted component (to1) depicts 14% as the orthogonal component. According to the findings of this comprehensive study, crude extracts from *S. albidoflavus* demonstrated varying abilities to impede phytopathogen growth and enhance root and shoot length in tested plants. Through untargeted metabolomics, we discovered numerous potential molecules with antagonistic activity against fungal phytopathogens among the top 10 significant metabolites with the highest absolute log2FC values. These include Tetrangulol, 4-Hydroxybenzaldehyde, and Cyclohexane. Additionally, we identified plant growth-regulating metabolites such as N-Succinyl-L-glutamate, Nicotinic acid, L-Aspartate, and Indole-3-acetamide. The KEGG pathway analysis has highlighted these compounds as potential sources of antimicrobial properties. The inhibitory effect of *S. albidoflavus* crude extracts on pathogen growth is primarily attributed to the presence of specific gene clusters responsible for producing cyclic peptides such as ansamycins, porphyrin, alkaloid derivatives, and neomycin. Overall, it is apparent that crude extracts from *S. albidoflavus* exhibited varying abilities to inhibit the growth of three phytopathogens and enhancement in both root and shoot length of tested plants. This research enhances our understanding of how secondary metabolites contribute to growth promotion and biocontrol, supporting ecosystem sustainability and resilience while boosting productivity in sustainable agriculture.

## 1 Introduction

Environmental sustainability is a global concern, prompting governments and industries to seek innovative solutions to mitigate the negative impact of human activities (Hailemariam and Erdiaw-Kwasie, 2023). With the world population projected to reach 9 billion by 2050, the agricultural sector faces significant challenges, including urbanization, climate change, diminishing soil fertility, and pathogen resistance (United Nations, 2022; Montagnini and Metzel, 2024). Pathogenic infections contribute to crop losses and food insecurity, and excessive fungicide use poses environmental threats (Savary et al., 2017; Le et al., 2022).

The Environmental Protection Agency (EPA) defines pesticides as substances used to prevent or destroy harmful organisms, including fungicides (Salazar et al., 2023). The agricultural sector is continuously evolving to reach the goal of of meeting sustainable development goals targets 2.1 and 2.2 to sustain the global population of 8 billion (FAO, 2024). Plant diseases are estimated to cause approximately $40 billion in annual global losses, directly and indirectly, impacting agricultural output and economic efficiency (Bregaglio et al., 2021; Li et al., 2023). Given the hazardous effects of chemical fertilizers and traditional fungicides like dimetachlone and mancozeb, minimizing their use is crucial. This shift will help manage plant pathogens through alternative methods, promoting sustainable agriculture (Mahanty et al., 2017).

Nowadays, researchers are now focusing on sustainable, cost-effective methods to manage plant diseases and enhance soil fertility without harming human health or the environment (Boukhatem et al., 2022). One promising approach to mitigate crop losses is the use of biological antagonistic agents, such as bacteria, for plant disease control (Sharma et al., 2017; Chen et al., 2020). The application of bacteria for the biological control of plant pathogens presents an environmentally friendly and promising approach that could reduce reliance on agrochemicals (Köhl et al., 2019; Li et al., 2023).

Biofungicides, which are microorganisms or their compounds toxic to plant pathogens, offer an environmentally friendly alternative to traditional fungicides (Salazar et al., 2023). Among the various biocontrol bacteria, endophytic bacteria produce essential compounds in abundance that are utilized by plants for their survival. These endophytic bacteria can safeguard plants from other phytopathogens due to their mutually beneficial relationships with host plants (Bataeva et al., 2023; Negi et al., 2023). For instance, *Thymus roseus*, a traditional Chinese medicinal herb used in this research study, is known as a valuable source of endophytic actinobacteria that have the ability to promote plant growth and development under challenging environmental conditions (Musa et al., 2020; Mohamad et al., 2022a).

Recent studies highlight the significance of plant-microbe relationships for sustainable agriculture, particularly in the discovery of bioactive small molecules with potential applications in sustainable agriculture (Fasusi and Babalola, 2021; Li et al., 2022; Nazari et al., 2023). Microbial secondary metabolites play a crucial role in interactions between organisms and the environment, serving as vital therapeutic substances in agriculture, they are commonly referred to as agro-antibiotics and antibiotics (Selim et al., 2021; Pan et al., 2019; Ghanem et al., 2022; Cai et al., 2023). There were commercial products comprised of *Streptomyces* species, such as Actinovate^®^ (Natural Industries, Inc.) and Mycostop^®^ (Verdara Oy), both used for the control of foliar and root diseases of various crops (Kim et al., 2019; Evangelista-Martínez et al., 2020). Additionally, several actinobacterial strains show promise as highly efficient biopesticides. For example, *S. avermitilis* ССМ 4697 produces avermectins, which are toxic to acari (Russian Federation (RF), 2000); *S. chrysomallus* Р-21 is active against fungal and viral phytopathogens (Russian Federation (RF), 2004); *S. hygroscopicus* subsp. TsKM V-4561 possesses fungicidal, bactericidal, and insecticidal properties (Russian Federation (RF), 2002).

One of the most widely used “omics” fields today is metabolomics. Metabolomics, the study of metabolites within a biological system, provides insights into biological processes and the correlation of biochemical activities involved in observed phenotypes by visualizing molecules that regulate biological interactions (Brunetti et al., 2018; Fu et al., 2022; de Medeiros et al., 2023). Metabolomics studies of plant microbiomes have provided additional insights into functional details beyond the genetic information of specific bacterial strains (Shen et al., 2023). Metabolomics, an emerging technology in recent years, can elucidate the physiological and pathological mechanisms by analyzing the overall changes in metabolites across different samples (Fu et al., 2022). Earlier reports have further shown that metabolomics analysis is commonly utilized to distinguish metabolite phenotypes and compare different metabolites, reflecting total metabolite information effectively. This approach allows for the analysis of similarities, differences, and changes in trends while providing comprehensive explanations (Yu et al., 2020; Iqrar et al., 2021; Oyedeji et al., 2021; Li et al., 2022). Specifically, metabolomics offers a holistic method for identifying and profiling the complete range of present metabolites at a specific time point (Alqahtani et al., 2022; Shen et al., 2023). Hence, the success of new antifungal strategies will require in the future cutting-edge technologies to identify novel biocontrol compounds. Therefore, metabolomics serves as a valuable tool in exploring the material basis of microbial co-culture, plant growth promotion, and disease prevention.

Our ongoing research demonstrates that *Streptomyces albidoflavus* (B24) promotes growth, and prevents disease, with co-culture enhancing these effects under challenging environmental conditions (Musa et al., 2020; Mohamad et al., 2022a). We hypothesize that these bacteria generate secondary metabolites capable of managing fungal pathogens and promoting plant growth. This study aims to understand the metabolite composition and changes when exposed to four fungal pathogens (*Fusarium oxysporum, Fusarium moniliforme, Verticillium dahliae Kleb,* and *Fusarium graminearum*) using metabonomic analysis technology through Q Exactive UPLC–MS/MS. Additionally, we examine the growth-enhancing and biocontrol impact of crude actinomycete extracts *in vitro*. This research provides the first scientific basis for an untargeted metabolomics approach to explore the antagonistic activity of crude extracts from *S. albidoflavus* (B24). It enhances our understanding of how secondary metabolites contribute to growth promotion and biocontrol, potentially aiding in ecosystem sustainability and agricultural productivity.

## 2 Materials and methods

### 2.1 Experimental materials

*Streptomyces albidoflavus* (B24) accession number (MN688247) was previously isolated and characterized by our research group from the Chinese medicinal herb *Thymus roseus* Schipcz obtained from the arid land in Ili and Tacheng of the Xinjiang Province, China (Musa et al., 2020). The standard fungal pathogens used in this study included *Fusarium oxysporum* (F1) ACCC37438*, Fusarium moniliforme* (F2) CGMCC (3.4269)*, Fusarium graminearum* (F3) CGMCC (3.3488), and *Verticillium dahliae* Kleb (F4) ACCC30308 which were provided by China General Microbiological Culture Collection Center (CGMCC).

### 2.2 Fermentation and crude extracts preparation of *Streptomyces albidoflavus* B24

A single colony of endophytic actinobacterial strain (B24) was well-cultured with ISP2 broth medium (monoculture). The flasks 500 mL were continuously cultivated in a shaker with 200 revolutions per minute (rpm) at 28°C for 21 days (Li et al., 2022). Each fungal strain (F1), (F2), (F3), and (F4) were grown in potato dextrose agar (PDA) plates for 6 days and the mycelial disc (5 mm) was transplanted into flasks 500 mL that were continuously cultivated in a shaker with 200 revolutions per minute (rpm) at 28°C for 21 days (monoculture). For Co-culture, a single colony of endophytic actinobacterial strain (B24) was cultivated in a modified liquid ISP2 medium and after 24 h of incubation, the mycelial disc (5 mm) of each fungus was transplanted into flasks 500 mL of modified media of ISP2 that were continuously cultivated in a shaker with 200 revolutions per minute (rpm) and 28°C for 21 days. When large-scale fermentation was performed, the fermentation broth was filtered through a Whatman No.1 filter followed by centrifugation at 10,000 rpm for 15 min to collect the supernatant. A three-step extraction was carried out on the supernatant in a 1:1 ratio (culture supernatant: ethyl acetate) (Supplementary Figure S1). The resulting crude extracts were then filtered and concentrated using a rotary vacuum evaporator at 40°C (N-1300, EYELA, Ailang Instrument Co., Ltd., Shanghai, China) as described by Zhang et al. (2022).

### 2.3 Evaluation of the antimicrobial activity of crude extract against fungal pathogens

To further investigate the inhibitory capacity of extracts from endophytic actinobacterial strains, we assessed their effect on spore germination of fungal strains (F1), (F2), (F3), and (F4) using a modified version of a previously described method (Abdelshafy Mohamad et al., 2020; Mohamad et al., 2022a). Briefly, fungal strains were cultivated in potato dextrose agar plates for 6 days. Then, mycelial discs with a diameter of 5 mm were transferred into the center of fresh PDA plates. Subsequently, filter paper discs with a 5-mm diameter, sterilized for 30 min at 121°C, were prepared using hole punches. Crude extracts containing bioactive metabolites were symmetrically applied onto four corners of each plate, positioned 2.5 cm away from the periphery of the plate. After wrapping all plates with parafilm, they were incubated at 26 ± 2°C for seven days and observed to assess pathogen inhibition. The percentage of growth inhibition was calculated by measuring the diameter of the inhibition zone by using the following formula (Mohamad et al., 2018):


$$\mathrm{Inhibition}\;\mathrm{rate}\;\left(\%\right)=\frac{F_{C}-T_{b}}{F_{C}-F_{0}}\times 100$$

where F_c_ is the fungal colony diameter on the control PDA base plate, T_d_ is the fungal colony diameter on the experimental PDA, and F_0_ is the diameter of the test fungus agar discs (approximately 5 mm). This experiment was conducted twice in triplicate.

### 2.4 Evaluation of growth-promoting effect by *Streptomyces albidoflavus* crude extracts

A monocot plant, *Echinochloa crus-galli* (L.) P. Beauv., and dicot species, *Amaranthus retroflexus*, were used as target plants for testing the growth-promoting effect of crude extracts (Tang et al., 2020; Wei et al., 2023). Seeds of the target plants were surface sterilized with 0.5% HgCl_2_. Isolated compounds from *Streptomyces albidoflavus* (B24) were dissolved in ethyl acetate to create solutions at concentrations of 5, 20, 100, and 500 μg/mL. Subsequently, the solutions were added to filter paper-lined Petri dishes (3 cm diameter). After the complete evaporation of the ethyl acetate, distilled water (2 mL) was added to each dish, followed by placing 10 seeds into it. Parallel controls were maintained by cultivating *E. crus-galli* and *A. etroflexus* seeds with distilled water. The Petri dishes were stored in darkness at a temperature of 25°C for 5 days while seedlings grew before measuring shoot and root lengths. Each treatment consisted of three replicates (*n* = 30). Statistical differences were analyzed using an ANOVA and Fisher’s LSD test at *p* < 0.05.

### 2.5 Extraction of metabolites

The LC/MS system utilized for metabolomics analysis consisted of a Waters Acquity I-Class PLUS ultra-high performance liquid tandem coupled with a Waters Xevo G2-XS QT of high-resolution mass spectrometer. The column employed was procured from Waters, specifically the Acquity UPLC HSS T3 column (1.8um 2.1*100 mm), and maintained at a temperature of 35°C with a flow rate set at 0.3 mL/min. In positive ion mode, mobile phase is a comprised of a 0.1% formic acid aqueous solution while mobile phase B featured a mixture of acetonitrile and 0.1% formic acid; negative ion mode shared the same composition for both mobile phases as in positive ion mode. A gradient elution program was executed according to the following schedule: 0–0.5 min, 90% A; 0.5–7 min, 90% A; 7–8.5 min, 0% A; 8.6 min, 0–90% A; 8.6–10 min, 90% A. The injection volume was 1 μL (Wang et al., 2023).

### 2.6 LC-MS/MS analysis

The Waters Xevo G2-XS QTOF high-resolution mass spectrometer is capable of collecting primary and secondary mass spectrometry data in MSe mode using the acquisition software (MassLynx v4.2; Waters Corporation, Milford, MA, United States). Each data acquisition cycle includes dual-channel data acquisition for both low collision energy and high collision energy simultaneously. The low collision energy is set at 2 V, while the high collision energy range varies from 10 to 40 V with a scanning frequency of 0.2 s for a mass spectrum. The parameters of the ESI ion source include: Capillary voltage—2000 V (positive ion mode) or-1500 V (negative ion mode); cone voltage - 30 V; ion source temperature—150°C; desolvent gas temperature-500°C; backflush gas flow rate-50 L/h; Desolventizing gas flow rate-800 L/h.

### 2.7 Data preprocessing and annotation

The raw data obtained from MassLynx V4.2 is analyzed using Progenesis QI software V3.0 for peak extraction, alignment, and other data processing procedures. This analysis was conducted with reference to the Progenesis QI software online METLIN database and Biomark’s self-constructed library for identification, and at the same time, ensuring that theoretical fragment identification and mass deviation all are within 100 ppm.

### 2.8 Data analysis

After normalizing the original peak area information with the total peak area, further analysis was conducted. Principal component and Spearman correlation analyses were used to assess sample repeatability within groups and quality control samples. The identified compounds were searched for classification and pathway information in KEGG (Kyoto Encyclopedia of Genes and Genomes) databases. According to the grouping information, the difference multiples were calculated and compared. *T*-tests were used to calculate the significance (*p*-value) of the difference for each compound. Additionally, Principal Component Analysis (PCA) was used (Zhou et al., 2018; Li et al., 2022; Oppong-Danquah et al., 2023). The R package ropls V3.19 were used for conducting OPLS-DA modeling, and the model’s reliability was verified through 200 permutation tests. The VI*p* value of the model underwent calculation via multiple cross-validation. A method which combined the differences multiples, *p*-values, and VIP values of the OPLS-DA model was employed to select the varied metabolites. The screening criteria consisted of FC > 1, P value<0.01, and VIP > 1. Differential metabolites with enriched significance in KEGG pathways were determined using a hypergeometric distribution test. Potential biomarkers were screened through MetaboAnalyst 4.0^1^ to identify potential metabolic pathways. Additionally, Spearman’s rank correlation analyses were conducted to explore the correlation between metabolites (Song et al., 2023).

## 3 Result

### 3.1 Comparative untargeted metabolomics analysis of *Streptomyces albidoflavus* metabolites

Microorganisms have the capacity to generate diverse metabolites under different ecological conditions. Our group previously isolated *S. albidoflavus* (B24) from wild populations of the Chinese medicinal herb *T. roseus*, obtained from arid areas of Xinjiang Province, China. The metabolomics evaluation of the interaction between *S. albidoflavus* and various fungal pathogens F1, F2, F3, and F4 extracts was examined using ultra-high-resolution mass spectrometry (Supplementary Table S1). This analysis utilized a widely targeted metabolomics approach in both positive (+) and negative (−) ion modes.

**Figure 1:**
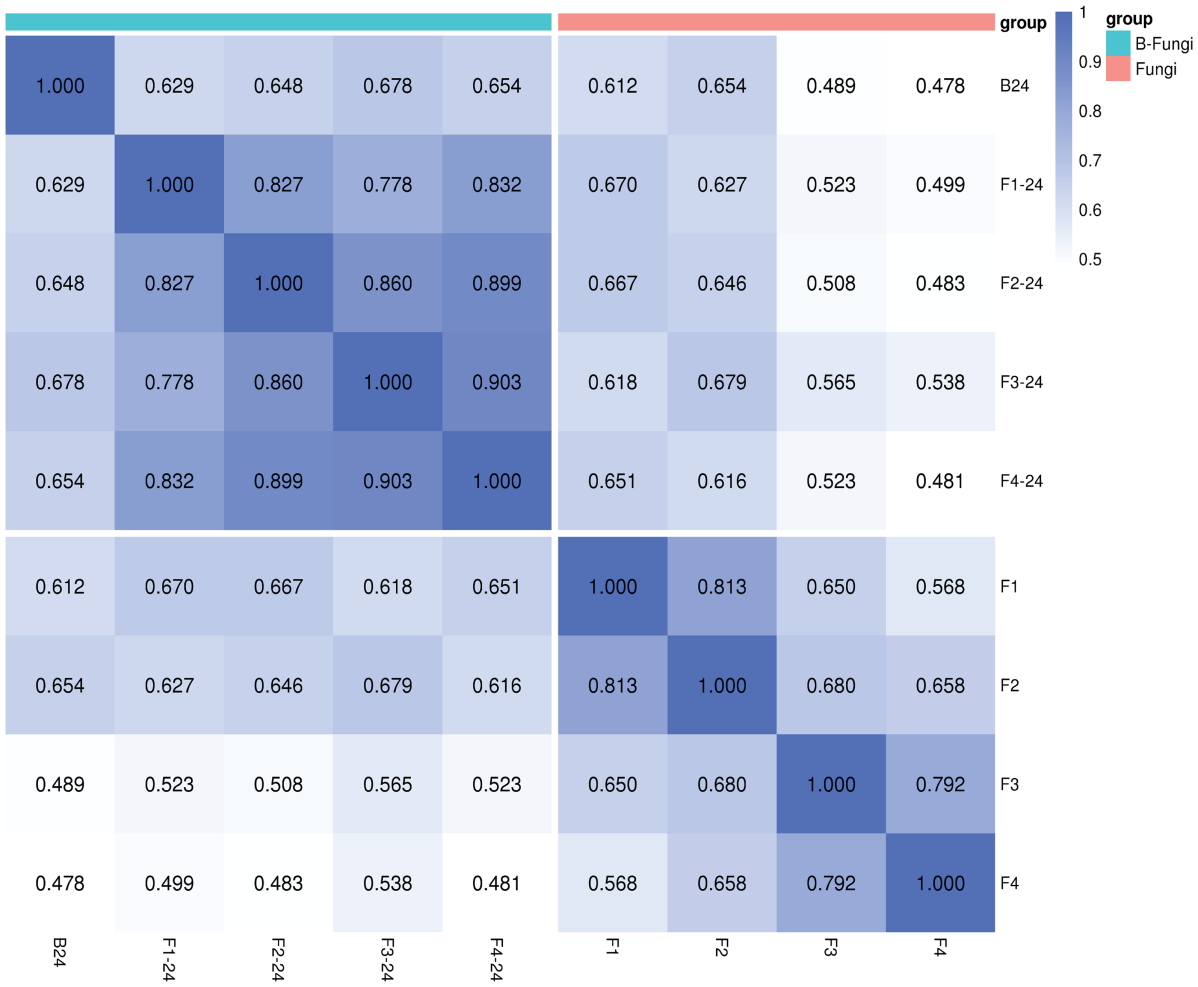
The Spearman rank correlation coefficient (SRC) of *Streptomyces albidoflavus* (B24) with fungal pathogens [*F. oxysporum* (F1), *F. moniliforme* (F2), *F. graminearum* (F3), and *V.dahliae* b (F4)].

The Spearman rank correlation coefficient (SRC) is utilized as an assessment metric for analyzing the correlation between samples within a group, where the intensity of the correlation coefficient is represented by the color depth. Our findings revealed that strain B24 demonstrated a stronger correlation coefficient when interacting with various fungi; for instance, 0.629, 0.648, 0.678, and 0.654 to *F. oxysporum* (F1), *F. moniliforme* (F2), *F. graminearum* (F3), and *V. dahliae* Kleb (F4) respectively (Figure 1). The correlation analysis indicated that the metabolites under different interaction conditions exhibited consistent repeatability within the group, thereby ensuring accuracy in our analysis.

### 3.2 Multivariate statistical analysis of inter-group differences

**Figure 2:**
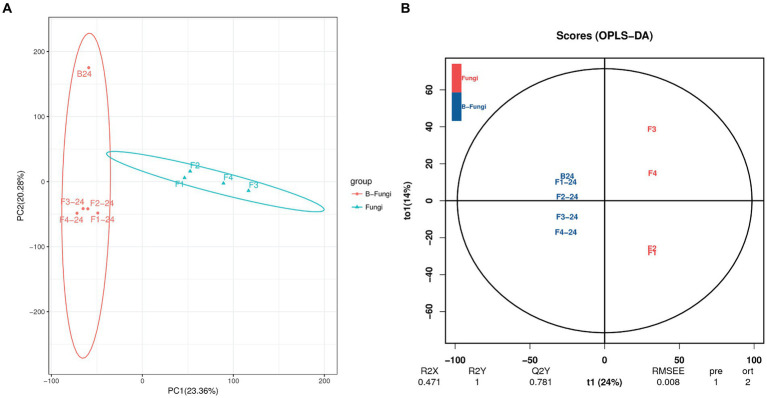
Multivariate statistical analysis of *S. albidoflavus* (B24) with fungal pathogens (*F. oxysporum* (F1), *F. moniliforme* (F2), *F. graminearum* (F3), and *V. dahliae* (F4)). **(A)** Principal Components Analysis (PCA); **(B)** orthogonal projections to latent structures-discriminant analysis (OPLS-DA).

The Principal Component Analysis (PCA) was employed to examine the disparities in the metabolome of *S. albidoflavus* between two groups using an unsupervised statistical method. The results depicted in Figure 2A demonstrate that PCA1 accounted for a 23.36% differentiation between the groups, while PCA2 accounted for a 20.28% distinction. The contrasts between the groups were more pronounced on PCA1, explaining the degree of separation between strain B24 and co-culture with different fungi. Furthermore, sample distribution along PC1 was primarily influenced by metabolite concentration in a positive direction. On the other hand, sample distribution along PC2 demonstrated distinct clustering both positively and negatively due to interactions within monoculture and co-culture with different fungi applied in this study. Consequently, PCA results indicated clear differences among various culture modes which align with previously analyzed variations in metabolites.

The method of principal component analysis mentioned previously can effectively extract key information but is less sensitive to weakly correlated variables. In contrast, partial least squares discriminant analysis orthogonal projections to latent structures-discriminant analysis (OPLS-DA) is a supervised pattern recognition multivariate statistical method that can efficiently identify differential metabolites by eliminating irrelevant effects. Figure 2B illustrates the OPLS-DA score plots, where the x-axis (t1) represents 24% as the predicted component (the difference between groups), and the y-axis (to1) depicts 14% as the orthogonal component (difference within the group). These score plots indicate significant separation among different comparison groups, suggesting notable differences in B24 monoculture’s metabolites compared with co-culture involving various fungi (Figure 2B). The data from this study revealed that both PCA and OPLS-DA analyses demonstrated significant alterations in metabolic profiles when comparing *S. albidoflavus* monoculture with co-culture conditions.

**Figure 3:**
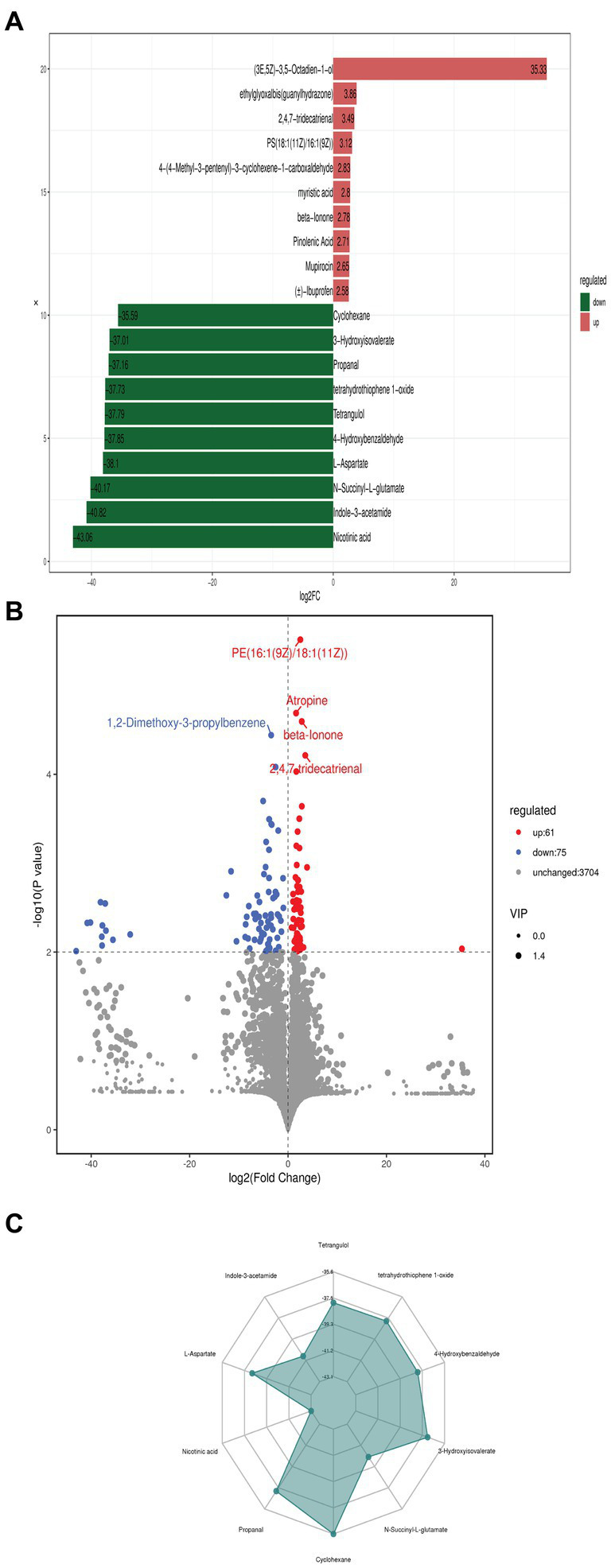
The fold change (FC) compares the variations in quantitative data of the top 10 up-regulated and down-regulated metabolites of *S. albidoflavus* monoculture with the co-culture **(A)**; Volcano Plot showed the differential metabolites at *p* < 0.01 **(B)**; Spider chart displayed the corresponding proportions for the quantitative values of different metabolites and identified the top 10 significant metabolites with the highest absolute log2FC values **(C)**.

The fold change (FC) compares the variations in quantitative data of the top 10 up-regulated and down-regulated metabolites in each group (Figure 3A). Our findings indicate that a total of 3,840 metabolites were identified, with 137 metabolites exhibiting significant differences between the two groups based on VIP >1, |FC| >1, and *p* < 0.01. Within these differential metabolites, there were 61 upregulated and 75 downregulated metabolites when comparing *Streptomyces albidoflavus* monoculture with the co-culture. Moreover, as shown in Volcano Plot (Figure 3B), it demonstrates a similar pattern regarding the differential abundance of metabolites across both groups along with statistical significance levels p < 0.01. Each dot portrayed on the volcano plot symbolizes individual metabolites, while their size represents variable importance in projection values from OPLS-DA model. On sorting by *p*-value, the figure highlights the top five significantly differing metabolites (Figure 3B). The spider chart displayed the corresponding proportions for the quantitative values of different metabolites and identified the top 10 significant antifungal and plant growth-regulated metabolites with the highest absolute log2FC values, such as Tetrangulol, Tetrahydrothiophene 1-oxide, 4-Hydroxybenzaldehyde, 3-Hydroxyisovalerate, N-Succinyl-L-glutamate, Cyclohexane, Propanal, Nicotinic acid, L-Aspartate, and Indole-3-acetamide (Figure 3C). As demonstrated in Figure 3C, larger values displayed more significant enrichment levels within specific pathogenic divisions. Additionally, the color depth of those points depicted relevant *p*-value correlations, significantly enriching those points.

### 3.3 Hierarchical clustering with heatmap and KEGG metabolic pathways

**Figure 4:**
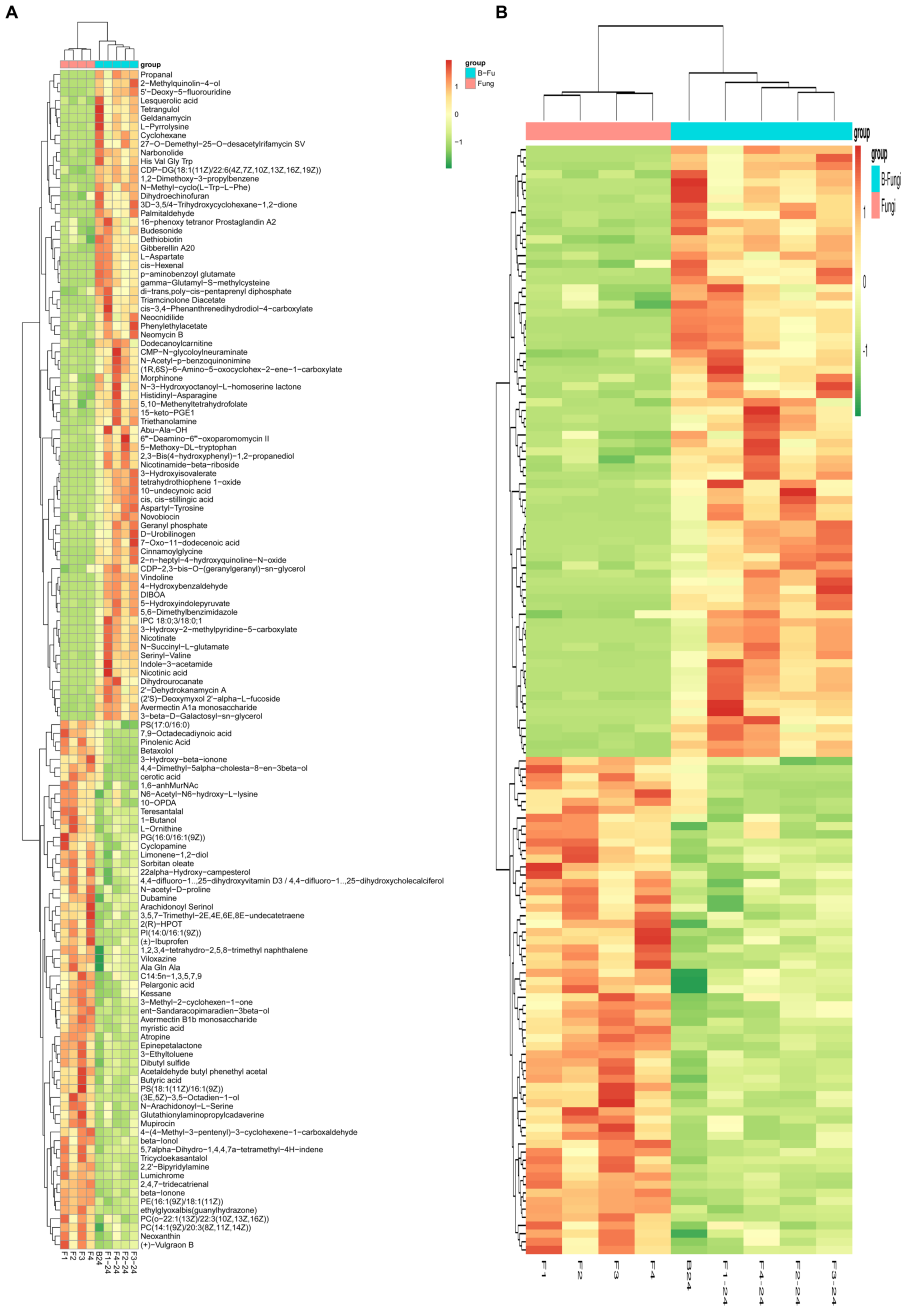
Heat map analysis of the identified metabolites of *S. albidoflavus* monoculture compared to those from co-culture. Hierarchical clustering of the identified 137 metabolites **(A)**; Standardized quantitative value of the Z-score for each metabolite categorized all metabolic spectra **(B)**.

For an overview of the metabolomic changes induced by *S. albidoflavus* monoculture compared to those from co-culture interactions with different fungi, a heat map was created using hierarchical clustering analysis to visualize the diverse concentrations of 137 metabolites and divided into groups. The results revealed that the overall metabolic the colors green, yellow, and red indicate higher abundance levels of metabolites in specific regions, suggesting substantial variations between the two groups (Figure 4A). Based on our results, the co-culture not only stimulated the production of new substances that were not detected in monoculture conditions but also has potential benefits for plant growth and biological control. For example, nicotinic acid, morphine, and tetrangulol could influence plant growth regulators or hormones (e.g., auxins, gibberellins) either directly or indirectly, promoting growth and providing biological control against pests and pathogens. On the other hand, some substances that were detected in monoculture conditions but not in co-culture conditions, such as L-glutamate and butyric acid, offer several benefits for plant growth, primarily through their positive effects on root development. These compounds could contribute to more resilient and sustainable agricultural practices. Additionally, several regions highlighted in green, red, yellow, and orange represent metabolites in higher abundances relative to the other regions with similar concentrations of different metabolites clustered together, demonstrating consistency among them. After hierarchical clustering, the standardized quantitative value of the Z-score for each metabolite categorized all metabolic spectra from *S. albidoflavus* monoculture compared to those from co-culture as shown in Figure 4B.

**Figure 5:**
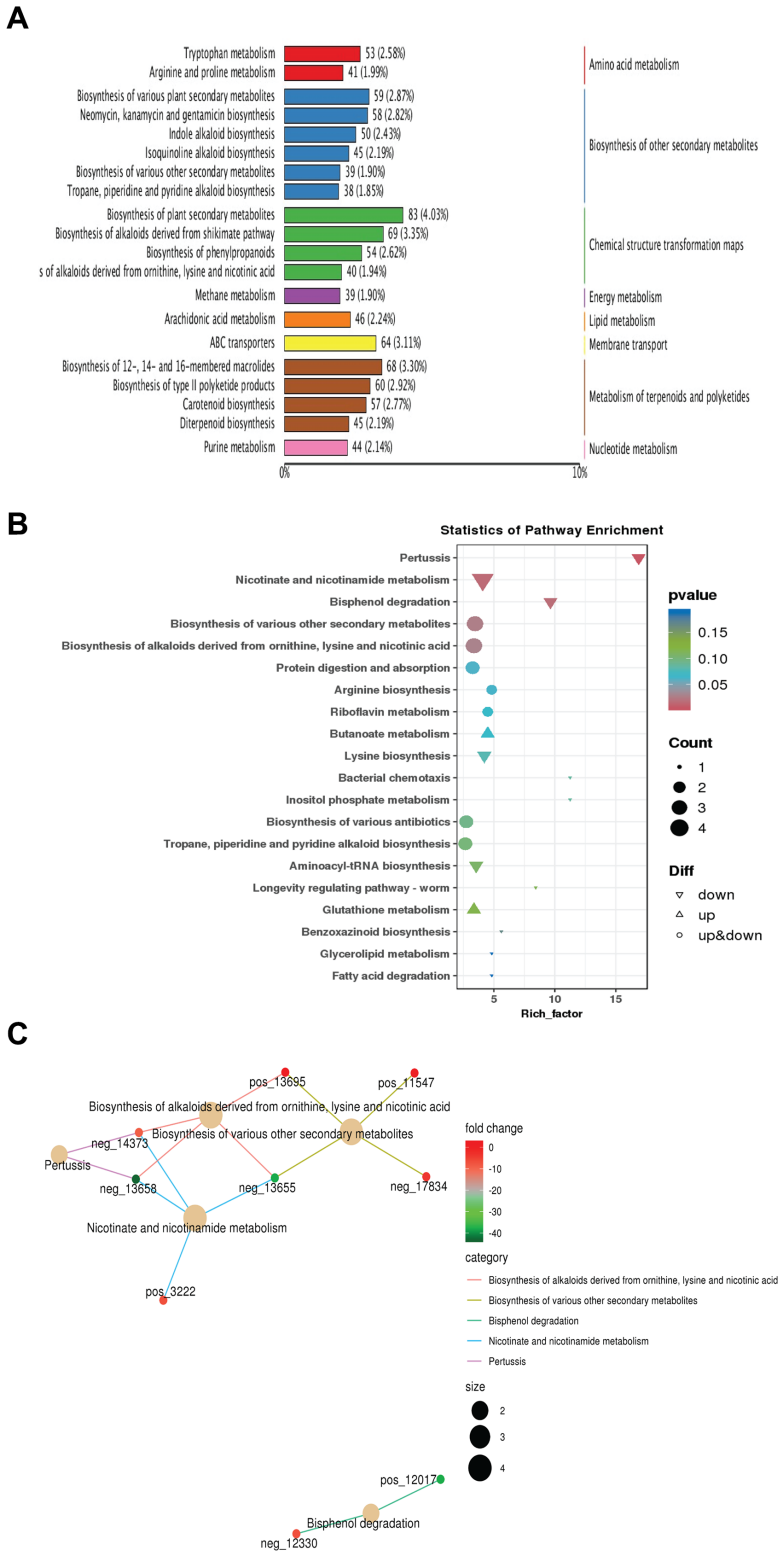
Analysis of KEGG metabolic pathways in case of monoculture and co-culture conditions of *S. albidoflavus*. The visualization of the results of top 20 metabolites with the highest number of differential annotations in the pathway **(A)**; KEGG pathway enrichment analysis **(B)**; Flod change (FC) of the top 5 differential metabolites annotated to the pathway at the value of log2 **(C)**.

To conduct enrichment analysis on the annotation outcomes of different metabolites interacting in organisms and forming various pathways, we utilized a hypergeometric test. The KEGG pathway serves as a bioinformatics tool aimed at comprehending high-level functions and utilities of biological systems by associating genomes with life and the environment. In this investigation, we employed the KEGG metabolic pathways database to label the top 20 metabolites with the highest number of differential annotations in the pathway (Figure 5A). Our findings indicated that these pathways were predominantly enriched in biosynthesizing various secondary metabolites involved in biological control and plant growth promotion activity such as Neomycin, Kanamycin, antibiotics up to 14.06%, alkaloids, and plant hormones up to 11.94% (Figure 5A). Subsequently, for further understanding of how biological pathways relate to proportionate metabolite levels; an enrichment factor analysis was conducted (Figure 5B). This analysis illustrated the ratio of differential up-and down-regulated annotated pathway-metabolites among all differential molecules compared to their overall representation across all metabolites related paths. For example, biosynthesis of various antibiotics was shown in up-and down-regulated-metabolites at a *p*-value of 0.15. In addition, the results showed that lysine biosynthesis was down-regulated. Lysine is an essential amino acid in plants, important for protein synthesis and serving as a precursor for glutamate, which regulates plant growth. On the other hand, the biosynthesis of arginine, an important and unique amino acid in plants, influences root growth. Fold change (FC) of the top 5 differential metabolites annotated to the pathway at a value of log2 such as biosynthesis of various secondary metabolites and alkaloids derived molecules as shown in Figure 5C. Overall, collectively considered results from our KEGG analyses imply that *S. albidoflavus* incorporates diverse metabolic pathways, especially involving multiple secondary compounds, amino acids, and chemical structure transformation maps metabolism.

### 3.4 Effects of *Streptomyces albidoflavus* B24 metabolites on seed germination

**Figure 6:**
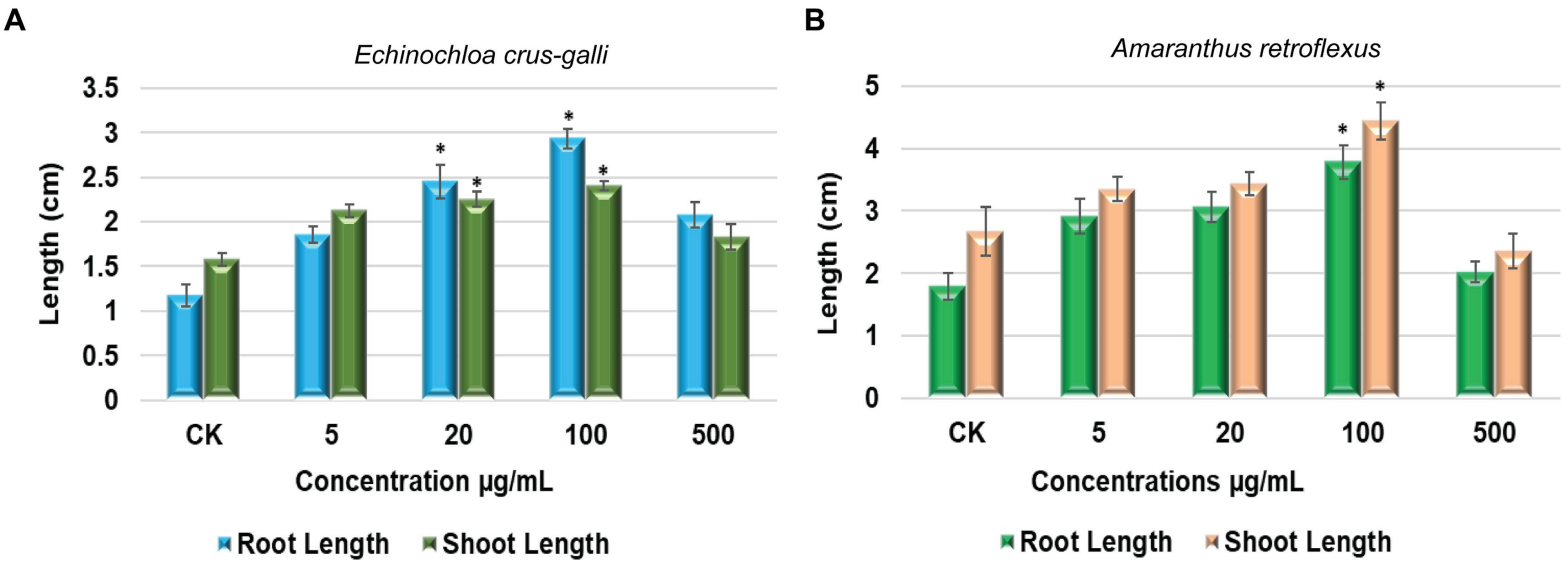
The impact of *S. albidoflavus* crude extracts on promoting the growth of root and shoot lengths **(A)** *E. crus-galli*; **(B)** *A. retroflexus*. Each value is the mean of five replicates and the standard error (SE) (*n* = 30). Stars represent a significant difference at *p* < 0.05 level according to Fisher’s LSD test.

The germination of seeds and the growth of seedlings in *Echinochloa crus-galli* P. Beauv., a monocot plant, as well as *Amaranthus retroflexus*, a dicot species, were selected to evaluate the impact of *S. albidoflavus* crude extracts on growth promotion. The concentrations tested ranged from 5 to 500 μg/mL (Figure 6).

Starting from a very low concentration (5 μg/mL), root length of *E. crus-galli* was notably enhanced by 68.45, 123.25, 166.42, and 88.75% when exposed to *S. albidoflavus* crude extracts at concentrations of 5, 20, 100, and 500 μg/mL, respectively (Figure 6A). As shown in Figure 6A, our results showed that shoot length exhibited a significant increase of 35.31, 43.25, 52.86% when treated with *S. albidoflavus* (B24) crude extracts at concentrations 5, 20, and 100 μg/mL, respectively, whereas when the concentration reached 500 μg/mL, the shoot growth was slightly promoted by 16.95%. In addition, the maximum significant increase (*p* ≤ 0.01) was observed in the root lengths by 166.42 and 52.86% for shoot length at 100 μg/mL.

Our results in this pilot study indicate that the growth of *A. retroflexus* roots was significantly enhanced by 53.17, 70.37, and 110.13% when treated with crude extracts of *S. albidoflavus* at concentrations of 5, 20, and 100 μg/mL, respectively, (Figure 6B). On the other hand, the effect was slightly weaker at a concentration of 500 μg/mL, resulting in only a 12.40% increase compared to other concentrations (Figure 6B). Conversely, shoot length increased by significant percentages 25.46, 28.79, and 66.33% in response to treatment with *S. albidoflavus* crude extracts at concentrations of 5, 20, and 100 μg/mL, respectively, but it inhibited growth by 11.16% at 500 μg/mL (Figure 6B). Overall, it is apparent that the most potent enhancement in both root and shoot length can be attributed to *S. albidoflavus* (B24) crude extracts at 100 μg/mL for both tested plants compared with the control.

### 3.5 Impact of *Streptomyces albidoflavus* metabolites to control plant disease

**Figure 7:**
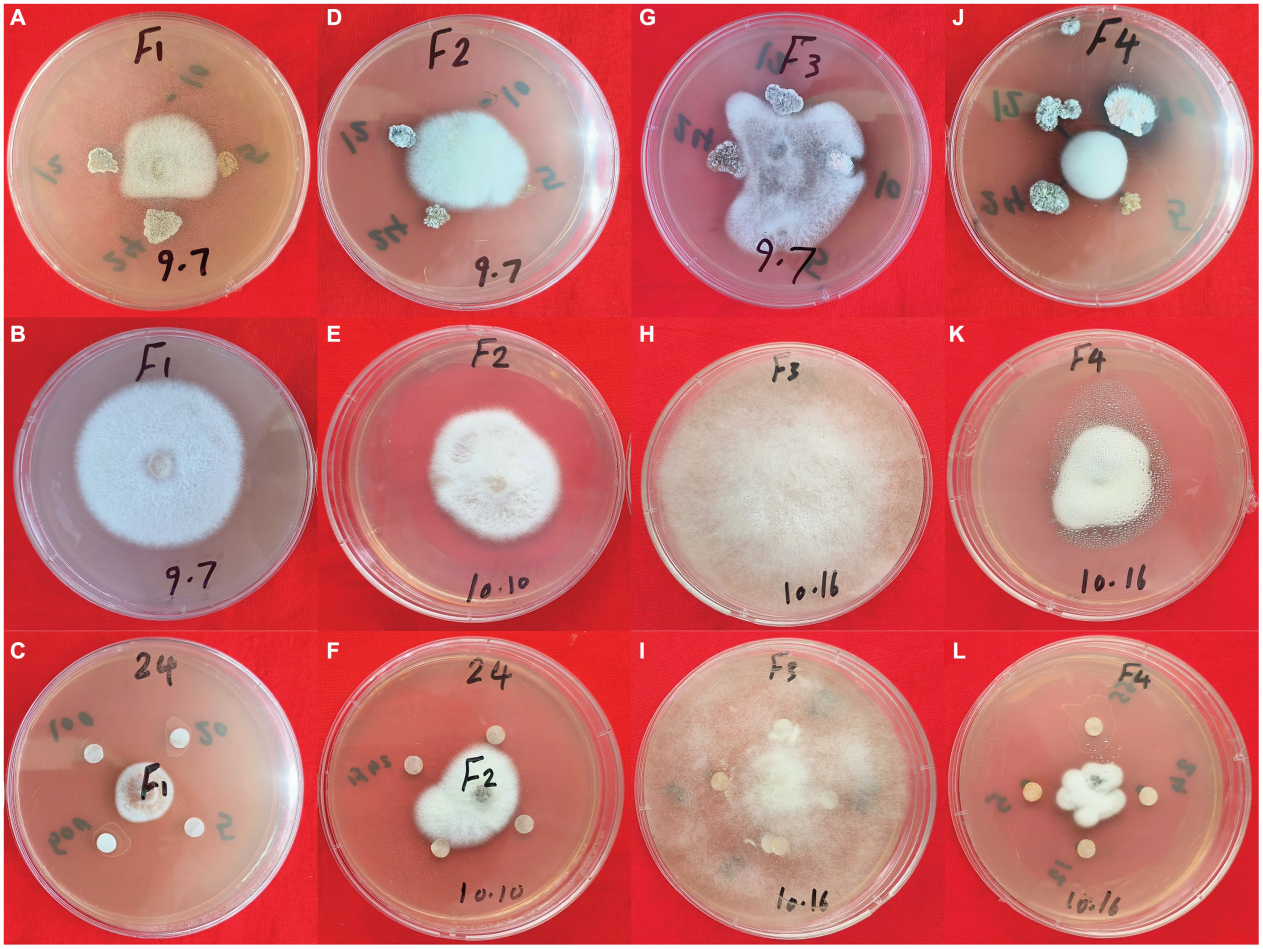
The antagonistic activity of *S. albidoflavus* crude extract against fungal pathogens. **(A–C)** *F. oxysporum* (F1); **(D–F)** *F. moniliforme* (F2); **(G–I)** *F. graminearum* (F3); **(J–L)** *V. dahliae* (F4).

To assess the suppressive effect of secondary metabolites from *S. albidoflavus* on the four fungal phytopathogens used in this investigation, a modified disc diffusion method was employed. The findings indicated that crude extracts from *S. albidoflavus* exhibited varying abilities to impede the growth of three phytopathogens used in this study F1, F2, and F4, with inhibition percentages ranging from 32.3 to 65.3%, as compared to the negative control (Figures 7A–F,J). Complete cessation of spore germination for the fungal phytopathogens together with observed morphological changes in fungal conidia were noted. However, no impact on *Fusarium graminearum’s* (F3) germination was observed (Figures 7G–I).

## 4 Discussion

The management of several severe fungal phytopathogens is exceedingly challenging, and the demand for products with minimal chemical residues continues to increase despite the use of larger quantities of fungicides (Carlucci et al., 2023). Microbial biotechnology represents a burgeoning scientific field with substantial potential applications, encompassing human health and plant protection (Fu et al., 2022; Gupta et al., 2022). Endophytes offer an effective approach to reduce plant diseases in agriculture through biological control of various phytopathogens by producing biologically active secondary metabolites (Hussain et al., 2020). *Streptomyces* species, a type of endophyte, have demonstrated effectiveness as biocontrol agents against various plant pathogens by producing bioactive compounds that can inhibit the mycelial growth of several fungal pathogens (Kaur et al., 2019; Joo and Hussein, 2022).

Based on the findings from our prior research, we propose that the endophytic actinobacterial strain B24, isolated from *Thymus roseus* roots and identified as *Streptomyces albidoflavus* by Musa et al. (2020) and Mohamad et al. (2022b) exhibiting both biocontrol and plant growth promotion activities. Within this context, the current study employs metabolomics technologies and bioinformatics approaches to explore the relationship between microbial metabolites and their interactions with various phytopathogens. In the study of microbial natural products, it is feasible to identify specialized metabolites produced by organisms under specific environmental conditions through qualitative and/or quantitative analysis (Genilloud, 2019). The Metabolomics approach facilitates the monitoring of chemical changes in biological systems metabolome, providing valuable insights into the impact of fungal interactions on the diverse metabolite structure of *S. albidoflavus* (Ghosh et al., 2021). The Spearman’s rank correlation was calculated to clarify these effects, revealing a strong positive correlation between *S. albidoflavus* and different fungi (*F. oxysporum* at 0.629, *F. moniliforme* at 0.648, *F. graminearum* at 0.678, and *V. dahliae* Kleb at 0.654) as illustrated in Figure 1. The network correlation analysis results indicated an evident relationship between the bacteria and fungi during microbial interactions that are specific to fungal species and exhibit high intragroup repeatability, ensuring analytical accuracy. This finding aligns with similar research conducted by Li et al. (2022).

In order to cover as many small molecules as possible in this investigation, negative electrospray ionization was also utilized due to the poor ionization of acidic and nonpolar compounds in positive electrospray ionization mode (ESI^+^) (Liu et al., 2023). In this study, the Principal Component Analysis (PCA) was employed to compare the metabolic profiles, including the presence and abundance of detected features, in both co-culture and monoculture samples (Ma et al., 2022). The results of the PCA revealed that the samples clustered into two distinct groups, indicating that there was no overlap between monoculture fingerprints and co-culture clusters (Figure 2A). This separation suggests that the datasets contained information enabling the discrimination of chemical composition in co-cultures from that in monocultures, suggesting that chemically mediated interactions influenced biosynthetic pathways for secondary metabolite production. Similar findings were reported by Shi et al., who observed the clustering of the samples in three groups, indicating that the co-culture fingerprints did not overlap with the two corresponding monoculture clusters (Shi et al., 2021).

To identify features that are specifically related to the co-culture compared to monoculture, the datasets of both cultures are analyzed using an S-plot. This plot uses the x-axis for a marker’s contribution to differences in grouping and the y-axis for confidence in this contribution. The MS features identified in the OPLS-DA analysis form two clusters: green markers on the right corner characterize the co-culture, while red markers on the left corner represent secondary metabolites upregulated and downregulated during chemical interactions with *Streptomyces albidoflavus* in monoculture (Figure 2B). These markers indicate shifts in biosynthetic pathways and metabolic regulation resulting from different chemical communication with bacterial interactions compared to fungi (Ghosh et al., 2020; Shi et al., 2021). However, it is essential to highlight that based on the VIP and *p* values of OPLS-DA, there were significantly different abundant metabolites. Therefore, chemical communication in this context represented a highly simulated interaction demonstrating the concept of chemical communications within the synthetic microbial community.

The differentially expressed metabolites were identified based on specific screening criteria: an absolute fold change (FC) of ≥1.5 and VIP of ≥1. The FC results for the top 10 up-regulated and down-regulated metabolites are shown in Figure 3A. Our results indicate that there were 61 upregulated and 75 downregulated differential metabolites showing significant differences between the two groups (Figure 3B). Additionally, the spider chart (Figure 3C) illustrated the proportions of different metabolite quantitative values, identifying the top 10 significant antifungal and plant growth-regulated metabolites with highest absolute log2FC values such as Tetrangulol, Tetrahydrothiophene 1-oxide, 4-Hydroxybenzaldehyde, 3-Hydroxyisovalerate, N-Succinyl-L-glutamate, Cyclohexane, Propanal, Nicotinic acid, L-Aspartate, and Indole-3-acetamide. The potential antifungal and plant growth promotion activity has been reported previously for some of them (Ahmad et al., 2021; Mendoza and Jaña, 2021; Yang et al., 2022; Fill, 2023; Liu et al., 2023; Tang et al., 2023).

According to the findings of the present study, a heat map was generated using hierarchical clustering analysis to illustrate the varied levels of 137 metabolites (Figure 4A). Additionally, the standardized quantitative Z-score value for each metabolite classified all metabolic spectra from *S. albidoflavus* monoculture in comparison to those from co-culture as displayed in Figure 4B. On the contrary, variations between green, yellow, and red colors indicated higher abundance levels of metabolites in specific areas, suggesting significant differences between the two groups. These results emphasize that red color represented higher concentrations of metabolites (Figure 4B). For instance, Geldanamycin is an antitumor agent belonging to the group of benzoquinone (Seo et al., 2023). L-pyrrolysine which a rare amino acids not found in all organisms (Meng et al., 2022). Dethiobiotin is a hexanoic acid and a member of the medium-chain fatty acids and important in biotin biosynthesis in microorganisms (Millan et al., 2022). While Indole-3-acetamide originates as a tryptophan derivative which serves as an intermediate compound during indole-3-acetic acid IAA biosynthesis process. The secretion of these compounds increased significantly when *S. albidoflavus* was grown under monoculture conditions compared with co-cultivation with other fungi (Tang et al., 2023) (Figure 4A). Conversely, morphinone is an oxidation product of morphine and has fungicidal and antifungal properties (Chu et al., 2024) was produced in high concentration in co-culture between B24 and F3 in comparison to monoculture (Figure 4A). The outcomes of our study align with the research conducted by Li et al., who indicated variations in metabolite types and quantities between two *Streptomyces* monoculture conditions and their co-culture (Li et al., 2022). Similarly, a recent publication by Millan et al. showed that the presence of the fungal strain *Botrytis cinerea* in co-culture conditions enhanced metabolic pathways involved in synthesizing and secreting several metabolites by *Metschnikowia pulcherrima*, including various alkaloids, antibiotics, and fatty acids (Millan et al., 2022).

Our research revealed that the endophytic actinobacterium *S. albidoflavus* is involved in numerous metabolic pathways, notably those associated with the biosynthesis of secondary metabolites such as Neomycin, Kanamycin, and various antibiotics. These metabolites, constituting up to 14.06% of the identified compounds, are recognized for their antifungal properties (Chang and Takemoto, 2014; Amin, 2019; Mani Chandrika and Sharma, 2020; Millan et al., 2022), alkaloid derivatives with antimicrobial activities (Othman et al., 2019; Chu et al., 2024), phytohormones (up to 11.94%), and terpenoids and polyketides (up to 2.92%) (Figure 5A). The ratio of differentially up-and down-regulated annotated pathway metabolites among all differential molecules was analyzed. This analysis, compared to their overall representation across all pathways related to metabolism, used an enrichment factor and fold change analysis (Figures 5B,C). The larger values reflected more substantial enrichment levels, while color depth indicated relevant *p*-values correlations, these results are in agreement with recent findings by Ma et al. (2022) and Wang et al. (2023).

Seed germination is a critical stage in the life cycle of a plant. Our results in this pilot study indicate that the growth of roots and shoot length of *E. crus-galli* and *A. retroflexus* was significantly *p* ≤ 0.01 enhanced when treated with crude extracts of *S. albidoflavus* at concentrations of 5, 20, and 100 μg/mL, respectively, (Figures 6A,B). Overall, it is apparent that the most potent enhancement in both root and shoot length can be attributed to *S. albidoflavus* (B24) crude extracts at 100 μg/mL for both tested plants compared with the control. This may be due to *S. albidoflavus* using multiple metabolite pathways to produce plant growth-regulated metabolites. According to the results of the current contribution as shown above (Figure 4C). The top 5 significant plant growth-regulated metabolites with the highest absolute log2FC values were N-Succinyl-L-glutamate, Cyclohexane, Nicotinic acid, L-Aspartate, and Indole-3-acetamide, they reported as essential metabolite for plant growth (Ahmad et al., 2021; Mendoza and Jaña, 2021; Yang et al., 2022; Fill, 2023; Liu et al., 2023; Tang et al., 2023). In addition, these findings are consistent with previous studies which revealed that *Streptomyces* strains showed plant growth-promoting activity by synthesizing biologically active secondary metabolites and significantly increased shoot and root length of *Salvia miltiorrhiza* (Du et al., 2022; Nazari et al., 2023).

**Figure 8:**
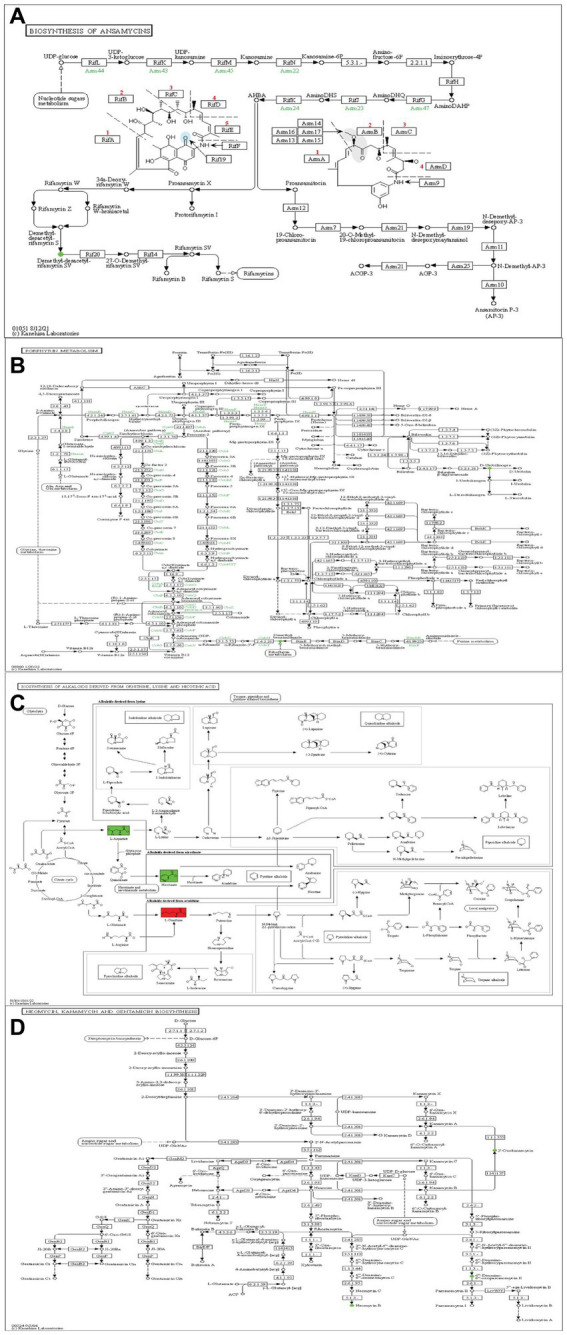
Metabolic pathways of *S. albidoflavus* **(A)** Biosynthesis of Ansamycins. **(B)** Porphyrin metabolism. **(C)** Biosynthesis of alkaloids drives. **(D)** Neomycin, Kanamycin, and Gentamicin Biosynthesis. Red color indicates a significant up-regulation metabolite and green one indicates a significant down-regulation metabolite.

*Fusarium* is a well-known genus of fungi that leads to substantial agricultural production losses, with an annual impact reaching up to 14% (Anand and Rajeshkumar, 2022). In China, around 3 million hectares of cotton crops suffer from *Verticillium* wilt, resulting in an annual yield loss of 10–30% (Mohamad et al., 2018). One key objective of this research was to enhance our comprehension of the interaction mechanism between *S. albidoflavus* and plant pathogens. The disc diffusion assay conducted in this study revealed that *S. albidoflavus* crude extracts inhibited the growth of phytopathogens F1, F2, and F4 by percentages ranging from 32.3 to 65.3%, as compared to control. The findings align with those previously documented by Newitt et al., who found that *Streptomyces* spp. effectively manage various diseases in cereal crops under different conditions (laboratory, greenhouse, and open-field) (Newitt et al., 2019). Likewise, Mamarasulov et al. (2023) reported that the ethyl acetate and methanol crude extracts of endophytic bacteria exhibit significant antimicrobial activity against *Pseudomonas aeruginosa*. Within this context, understanding the mechanisms and metabolites responsible for the antifungal strategies employed by *S. albidoflavus* crude extracts against fungal pathogens F1, F2, F3, and F4 is of great interest. Through untargeted metabolomics, many potential molecules with antagonistic activity against fungal phytopathogens were discovered such as Tetrangulol (Charousova et al., 2018), 4-Hydroxybenzaldehyde (Ahluwalia et al., 2016), and Cyclohexane (da Silva et al., 2023). According to the findings of this comprehensive study, the KEGG pathway maps have been identified as a potential source of compounds with antimicrobial properties. The inhibitory effect of *S. albidoflavus* on pathogen growth is primarily attributed to the presence of specific gene clusters responsible for producing cyclic peptides such as natamycin (Nong et al., 2020), porphyrin (Kodedová et al., 2023), alkaloid derivatives (Chu et al., 2024), and neomycin (Takemoto et al., 2022). These compounds exhibit potent antimicrobial activities (Figures 8A–D).

## 5 Conclusion

Our study identified 3,840 metabolites, with 137 showing significant differences between *Streptomyces albidoflavus* B24 monoculture and co-culture conditions. Among these, 61 metabolites were upregulated, and 75 were downregulated. The metabolites exhibited consistent repeatability in interactions with various fungi, showing correlation coefficients from 0.629 to 0.678. PCA analysis indicated 23.36% differentiation (PCA1) and 20.28% distinction (PCA2) between groups, with significant separations observed in OPLS-DA plots. We identified the top 10 significant antifungal and plant growth-regulating metabolites, including Tetrangulol, 4-Hydroxybenzaldehyde, and Indole-3-acetamide. Crude extracts of *S. albidoflavus* significantly enhanced root and shoot growth of *E. crus-galli* and *A. retroflexus*, particularly at 5, 20, and 100 μg/mL concentrations. The extracts also inhibited the growth of phytopathogens F1, F2, and F4, with inhibition rates from 32.3 to 65.3%, but had no impact on *F. graminearum* (F3). Our research provides a scientific basis for the first time of an untargeted metabolomics approach for the exploration of *S. albidoflavus* crude extracts providing valuable insights into monitoring of chemical changes in biological systems metabolome and enhancing our comprehension of how secondary metabolites contribute to both growth promotion and biocontrol effects, potentially aiding in ecosystem sustainability and resilience amidst climate change, and helped to increase the productivity and achieve agricultural sustainability in the green agriculture system.

## Data Availability

The original contributions presented in the study are included in the article/Supplementary material, further inquiries can be directed to the corresponding authors.

## Footnotes

^1^http://www.metaboanalyst.ca/
