# Supplementary figures and images for: Metabonomic analysis to identify exometabolome changes underlying antifungal and growth promotion mechanisms of endophytic Actinobacterium Streptomyces albidoflavus for sustainable agriculture practice
Source: Front Microbiol. 2024 Aug 30;15:1439798. doi: 10.3389/fmicb.2024.1439798 (PMC11393692; doi:10.3389/fmicb.2024.1439798)

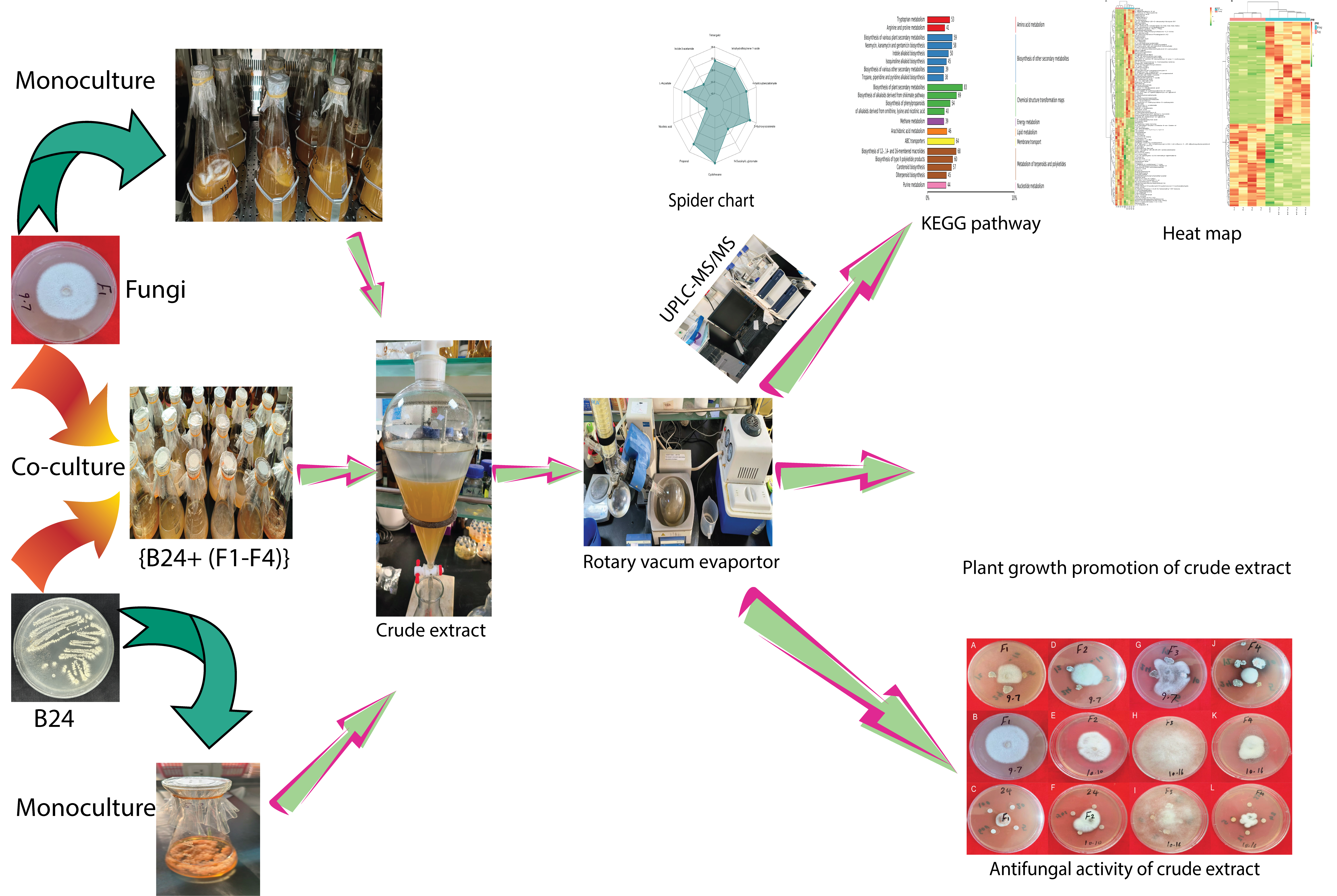

Supplement: Supplementary file 2 [file Image_1.TIF]
